# Successful hybrid surgery for ileal conduit stomal varices following oxaliplatin-based chemotherapy in a patient with advanced colorectal cancer

**DOI:** 10.1186/s40792-020-01021-6

**Published:** 2020-09-29

**Authors:** Hideo Uehara, Hirofumi Kawanaka, Tomonori Nakanoko, Masahiko Sugiyama, Mitsuhiko Ota, Yohei Mano, Keishi Sugimachi, Masaru Morita, Yasushi Toh

**Affiliations:** 1 Department of Gastroenterological Surgery, National Hospital Organization Kyushu Cancer Center, Notame 3-1-1, Minami-ku, Fukuoka, 811-1395 Japan; 2 Clinical Research Institute and Department of Surgery, National Hospital Organization Beppu Medical Center, 1473 Uchikamado, Beppu, Oita 874-0011 Japan; 3 Department of Hepatobiliary-Pancreatic Surgery, National Hospital Organization Kyushu Cancer Center, Notame 3-1-1, Minami-ku, Fukuoka, 811-1395 Japan

**Keywords:** Ectopic varices, Colorectal cancer, Hybrid surgery, Sinusoidal obstruction

## Abstract

**Background:**

Ectopic variceal bleeding is a rare but life-threatening complication of portal hypertension (PH). Oxaliplatin-based chemotherapy for colorectal cancer (CRC) is associated with sinusoidal obstruction syndrome of the liver, which can lead to PH.

**Case presentation:**

Here, we report a successful hybrid surgery that included intraoperative obliteration of ileal conduit stomal varices (ICSVs) for a 66-year-old woman with CRC and liver metastasis that had been treated multimodally during the previous 4 years, including 17 courses of oxaliplatin-based chemotherapy. She was admitted to our hospital for massive hemorrhage from an ileal conduct stoma. Image findings showed ICSVs as a part of portosystemic shunt, which were afferently supplied from the superior mesenteric vein (SMV) and drained by the numerous cutaneous veins connected to the left femoral vein. Obliteration of the stomal varices by interventional radiologic techniques alone was inappropriate because of difficulties of cannulating the efferent cutaneous veins. We, therefore, performed hybrid surgery for the ICSV, which included cannulation into the SMV branch and antegrade obliteration of the varices with a 5% solution of ethanolamine oleate with iopamidol under blocking the SMV flow, using a vascular clip and ligation. Hemorrhage in her ileal conduit stoma disappeared completely.

**Conclusion:**

Customized treatment of ectopic varices should be based on their precise vascular anatomy; hybrid surgery with intraoperative angiography is an alternative treatment for ectopic varices such as ICSV.

## Introduction

Varices are common complications in patients with portal hypertension (PH) [1–3]. They are typically located in the esophagus or stomach; varices in other sites, including the duodenum, jejunum, ileum, colon and rectum and stomas, are considered ectopic varices [2]. Ectopic varices in the small intestine and stoma are usually associated with adhesions of the small or large intestine to the abdominal wall as a result of prior surgeries.

Oxaliplatin-based chemotherapy is a standard treatment for advanced unresectable colorectal cancer (CRC) [4, 5]. However, as multidisciplinary treatment for CRC has progressed due to the development of surgery, radiotherapy and chemotherapy, survival of patients with CRC has extended, diverse adverse events, such as oxaliplatin-induced liver damage due to sinusoidal obstruction syndrome, have been brought to the surface [6, 7]. Oxaliplatin-based chemotherapy can cause thrombocytopenia, splenomegaly or portosystemic collateral vessels as a manifestation of PH. Ectopic variceal bleeding is a serious complication that requires special attention, especially in patients with CRC who have undergone prior surgeries, even those with no history of chronic liver disease.

Managing ectopic variceal bleeding should be decided considering the bleeding site, the cause of bleeding and general condition, which is difficult and still unclear. The treatment of ectopic variceal bleeding requires diverse treatments, including the trans-jugular intrahepatic portosystemic shunt (TIPS) procedure, transhepatic embolization, direct percutaneous embolization, balloon-occluded retrograde transvenous obliteration (BRTO), and other surgeries [8–11]. Here we report a case of ectopic variceal bleeding from an ileal conduit stoma, successfully treated with hybrid surgery that included intraoperative obliteration of the varices and their feeding and drainage veins.

## Case report

**Fig. 1:**
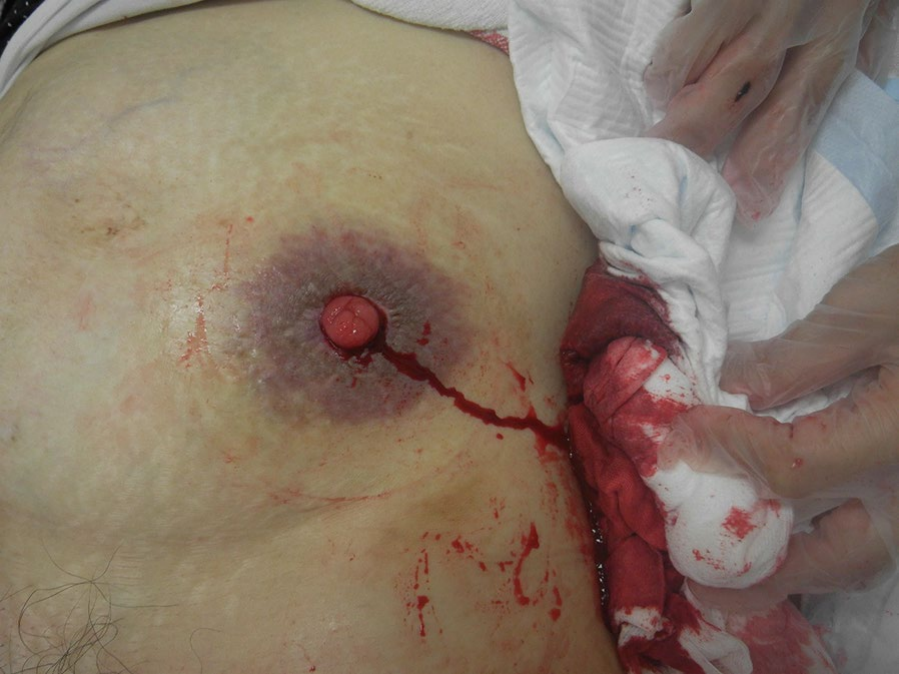
Ileal conduit stoma with bleeding and purplish peri-stomal discoloration

A 66-year-old woman was admitted to our hospital for marked hemorrhage in her ileal conduit stoma (Fig. 1). She had previously received many blood transfusions. Eight years before this admission, she had been diagnosed with rectal cancer that had invaded her small intestine, uterus and urinary bladder, and had metastasized to her liver. She underwent a colostomy and intestinal bypass surgery to improve her ileus. After this surgery, she received nine sessions of oxaliplatin-based chemotherapy (mFOLFOX6) plus bevacizumab. We then performed a low anterior resection of her rectum, combined with resections of her uterus, both ovaries, fallopian tubes, and urinary bladder, and a further urinary diversion by ileal conduit. After this second surgery, she underwent eight cycles of mFOLFOX6 plus bevacizumab. We then performed a partial liver resection, and closure of the colostomy, which was followed by 6 months of adjuvant chemotherapy with capecitabine, after which she had no recurrence for 8 years. However, in the last 4 years, she had been admitted several times for bleeding from her ileal conduit stoma.

**Fig. 2:**
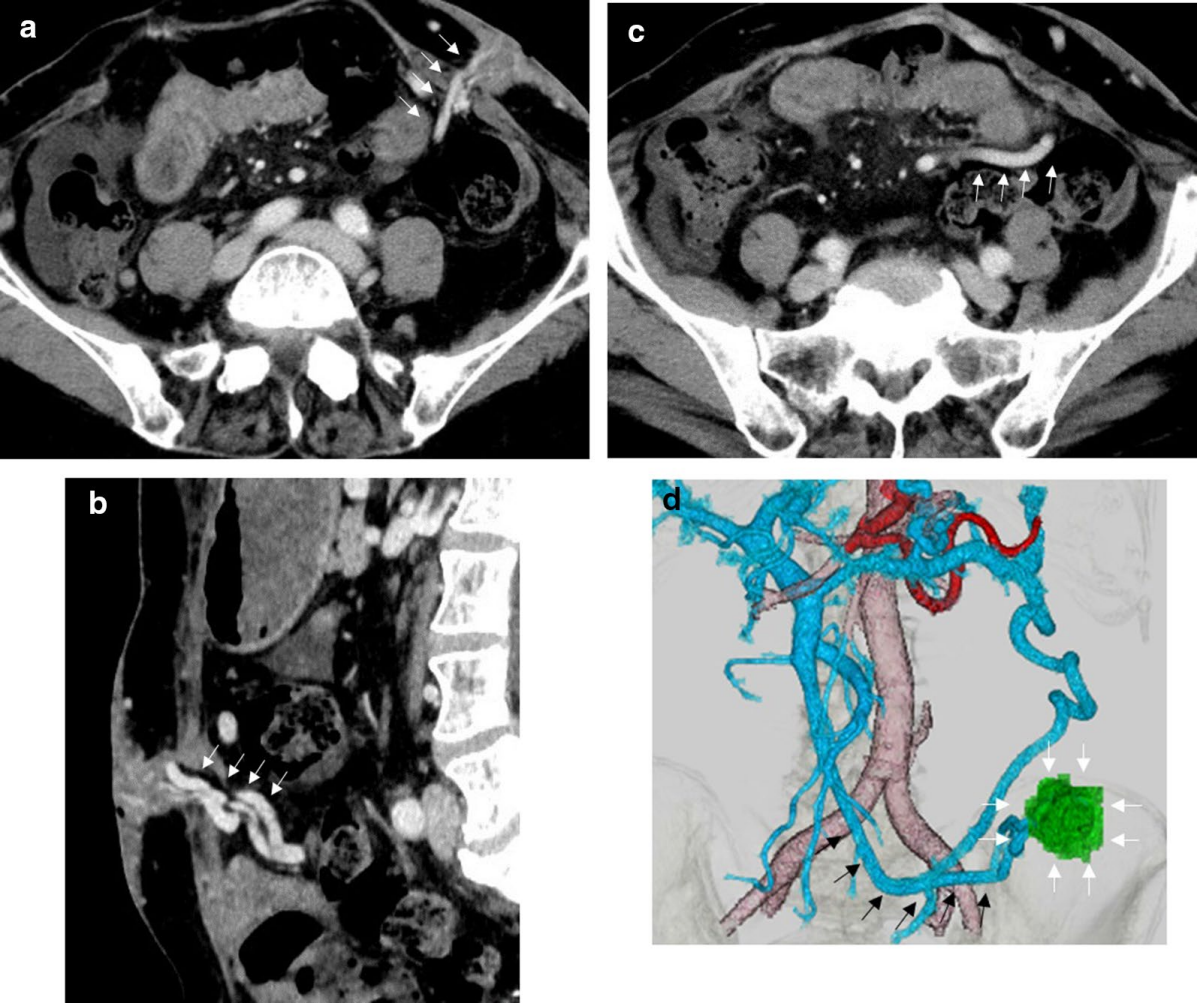
Abdominal CT imaging. **a** (horizontal slice), **b** (sagittal slice) Ectopic varices and collateral vessels at the ileal conduit stoma (arrow). **c** The varices were fed by superior mesenteric vein (arrow). **d** Three-dimensional reconstruction showed the ileal conduit stomal varices (white arrow) fed by the superior mesenteric vein (black arrow)

On her last admission, she had a Child–Pugh score of 8 (Child–Pugh class B) and her laboratory test results included hemoglobin: 5.6 g/dL, albumin: 2.4 g/dL, platelets: 106,000/μL, and prothrombin time: 82%. Contrast-enhanced computed tomography (CT) showed ileal conduit stomal varices (ICSVs) had developed as a part of the portosystemic shunt, which were supplied from the superior mesenteric vein (SMV) and drained by the numerous cutaneous veins connected to the left femoral vein (Fig. 2). BRTO was not appropriate because of difficulties of cannulating numerous cutaneous veins; and percutaneous transhepatic catheterization has the risks linked to puncturing the portal veins. Therefore, we selected hybrid surgery with intraoperative obliteration for the ICSV.

**Fig. 3:**
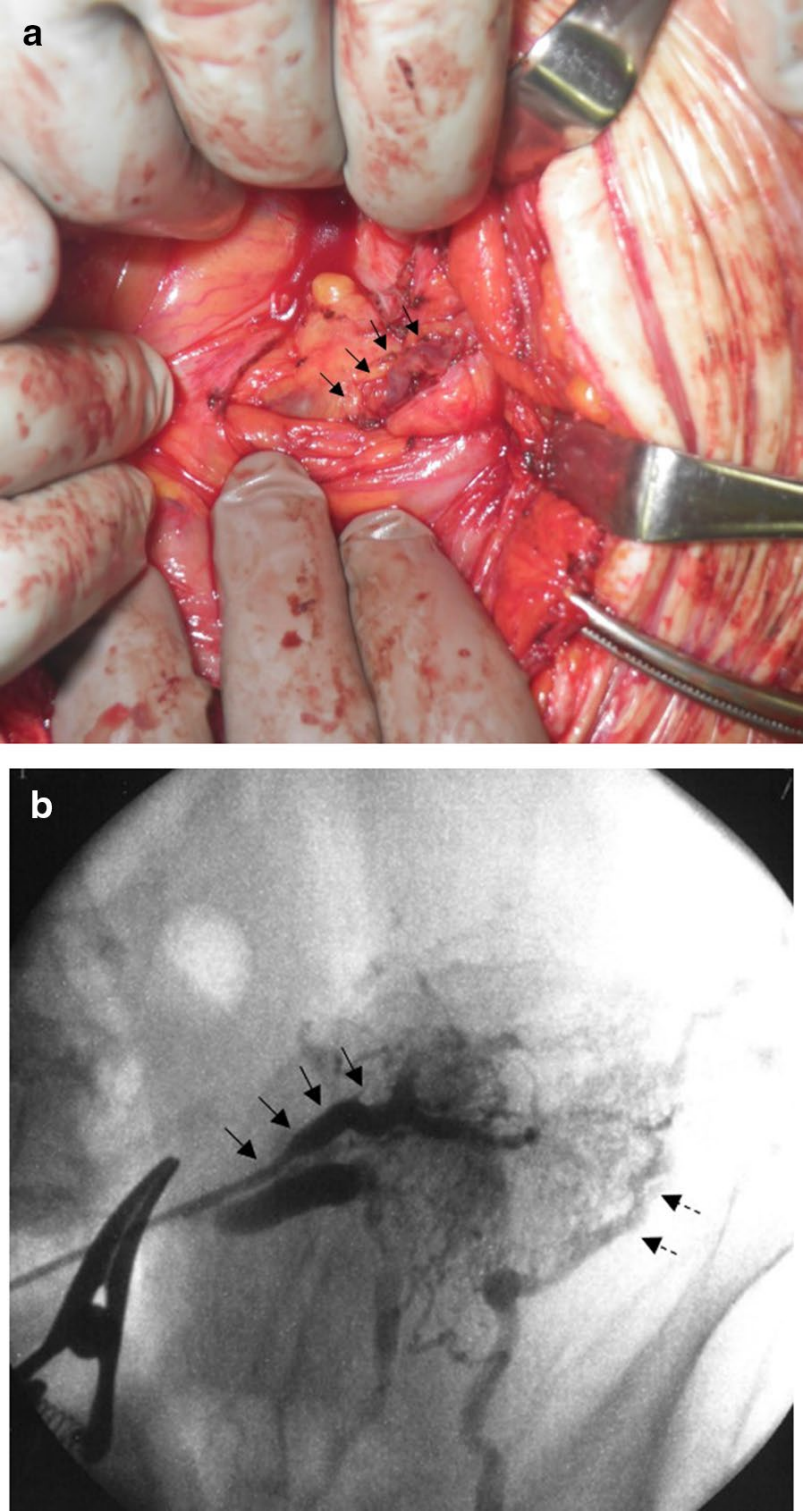
**a** After dissecting the dense peritoneal adhesion, we found dilated and tortuous vessels in the mesentery of small intestine. **b** Angiography showed sufficient opacification of stomal varices, including the two SMV feeding veins (arrow), which were numerous cutaneous veins around ileal conduit stoma that communicated the left femoral vein (arrow dotted)

Under general anesthesia, the patient was placed in a lithotomy position. The skin was incised on the upper and lower abdomen. Adhesions around her small intestine and ileal conduit stoma were very severe. After dissecting a dense peritoneal adhesion, we detected one of the two SMV branches in the ileal conduit mesentery, which were the ICSVs’ feeding veins. We tried to measure the portal vein pressure, but the adhesion in the abdominal cavity was severe and it is difficult to cannulate the SMV to the portal vein. We introduced a 4-Fr sheath (Create Medic, Tokyo, Japan) into the vessel (Fig. 3a). As angiography did not initially show sufficient opacification of the stomal varices, we blocked the blood flow of the feeding vein using a proximally placed vascular clip on the SMV. Angiography then showed sufficient opacification of the stomal varices, including the two SMV feeding veins and the numerous cutaneous drainage veins (Fig. 3b). While blocking the afferent blood flow to the stomal varices, we injected a 5% solution of ethanolamine oleate with iopamidol in an anterograde manner, with sufficiently opacified stomal varices and their feeding and drainage veins; the two SMV branches were then ligated. The lesion was treated with 15 mL of 5% solution of ethanolamine oleate with iopamidol.

**Fig. 4:**
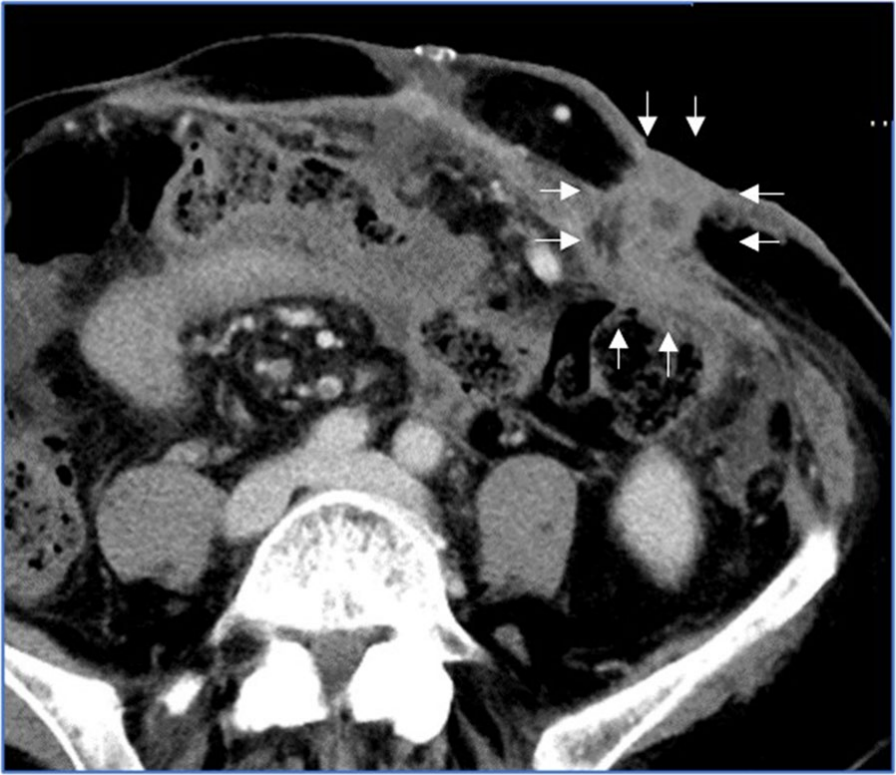
Abdominal CT imaging 7 days after the hybrid surgery. The feeding veins were occluded and the ileal conduit stomal varices had disappeared

Seven days after the hybrid surgery, CT showed that the ICSV had disappeared, and the feeding and drainage veins were occluded (Fig. 4), but partial portal vein thrombosis had developed in the SMV. We administered anticoagulation therapy with antithrombin-III concentrates and danaparoid sodium [12, 13], and that portal vein thrombosis disappeared by 14 days after the surgery. She was then discharged from our hospital. She has had no bleeding from her ileal conduit stoma, and no worsening of her portal thrombosis or liver function for 1 year after the hybrid surgery.

## Discussion

Hemorrhage from gastrointestinal variceal bleeding is the most serious and life-threatening complication among patients with PH [1–3]. Typically, gastrointestinal varices are located at the gastroesophageal junction; varices located outside the esophagus and stomach are, therefore, considered ectopic varices. Ectopic variceal bleeding is rare, and is an uncommon cause of gastrointestinal hemorrhage in portal hypertensive patients; it is difficult to diagnose and treat [2]. ICSVs are a rarely reported type of ectopic varices in patients who develop PH after urinary diversion [14–18].

To our knowledge, hybrid surgery for ICSV has never been reported before. Hybrid surgery that combines angiography during surgery provides much information to complete the shunt surgery, especially in cases with history of poly-surgery, obesity and the complicated vascular anatomy of ICSV. Here we report a case of ICSV, for which conventional treatment would have been challenging because of repeated massive bleeding over a long time, but was successfully treated with hybrid surgery.

PH is caused by liver cirrhosis, which may be caused by chronic hepatitis B or C, alcoholic hepatitis, non-alcoholic steatohepatitis, autoimmune hepatitis, or idiopathic PH [19]. In this case, the patients had no history of liver disease, so liver damage induced by oxaliplatin-based chemotherapy for CRC is thought to be the cause of her PH. Oxaliplatin-based chemotherapy is a standard systemic chemotherapy for advanced unresectable recurrent CRC [4, 20]. However, liver injury due to sinusoidal obstruction syndrome is a reported side effect of oxaliplatin [6, 7]. Details of the relationship between duration of oxaliplatin administration and the onset of liver damage are unclear, but liver damage apparently becomes more likely as the number of chemotherapy courses increase; therefore, more careful follow-up is required for oxaliplatin patients, especially those treated for six courses or more [21, 22]. This patient developed splenomegaly, thrombocytopenia, and collateral formation around the time of her 9th mFOLFOX6 plus bevacizumab course before her second surgery.

Ectopic varices in the small intestine and stoma are usually associated with adhesions to the abdominal wall, resulting from prior operations, so accurate diagnosis is difficult, and often is not found until well after onset. Recent advances in multi-detector row CT (MDCT) have made detection of ectopic varices easier [23, 24]. Three-dimensional angiography with MDCT is useful for identifying collateral circulation of ectopic varices and determining optimal treatment methods. In this case, MDCT showed the ICSV was fed by the SMV and drained into the left femoral vein. Patients with gastrointestinal bleeding and PH should receive MDCT to diagnose ectopic variceal bleeding, especially for patients with histories of prior surgery.

Methods to treat ectopic intestinal varices, including TIPS, BRTO, surgical treatment and medical treatment, have been widely reported, but the standard treatment has not been established yet. Treatment for ectopic varices should be determined after diagnosing the location and clinical symptoms, and by fully considering the risks and benefits of the treatment method. According to previous reports, nine cases of ICSV associated with portal hypertension after urinary diversion have been reported. In eight of all cases, endovascular treatment was performed and most of the endovascular treatment was TIPS [15, 17, 25–31]. TIPS—which uses a percutaneous catheter to create a short circuit between the portal and hepatic veins in the liver—has been widely used in Western countries for reducing the pressure of portal vein as a treatment for PH, but seems to be unsatisfactory for ectopic varices, with a reported 21–37% rebleeding rate [8, 32], arguably because ectopic varices are formed as a part of portosystemic shunt, so the pressure of portal vein is mostly lower in patients with ectopic varices than in those with esophageal varices [10, 33]. TIPS may also cause complications, such as exacerbation of hepatic encephalopathy and stent occlusion. BRTO has been widely used to treat gastrointestinal varices and hepatic encephalopathies associated with portosystemic shunts [9–11, 32, 33], and is minimally invasive, but may cause esophageal varices to develop or worsen, owing to post-treatment increase in portal pressure, and have difficulty in cannulating the variceal drainage veins. In this case, the ICSV drainage vein was narrow and meandering, which made retrograde cannulation difficult. We, therefore, selected hybrid surgery to treat the ICSV, identifying the variceal feeding vessels and obliterating the ethanolamine oleate with iopamidol while blocking venous flow to the stomal varices. If no adhesions are found around the ileum, trans-ileocecal antegrade obliteration with a balloon catheter is an option for obliterating the varices.

## Conclusion

In conclusion, therapeutic strategy of ectopic varices should be based on their precise vascular anatomy to avoid fail to obliterate or prevent recurrence of ectopic varices. Hybrid surgery that combines angiography provides the information about the vascular anatomy of ectopic varices including the collateral circulation intraoperatively and can be an alternative treatment of ectopic varices such as the ICSV.

## Data Availability

Data sharing is not applicable to this article, as no datasets were generated or analyzed during the current study.

## Abbreviations

PH: Portal hypertension
CRC: Colorectal cancer
ICSVs: Ileal conduit stomal varices
SMV: Superior mesenteric vein
TIPS: Trans-jugular intrahepatic portosystemic shunt
BRTO: Balloon-occluded retrograde transvenous obliteration
CT: Contrast-enhanced computed tomography
MDCT: Multi-detector row CT
